# The Immunologic Basis for Severe Neonatal Herpes Disease and Potential Strategies for Therapeutic Intervention

**DOI:** 10.1155/2013/369172

**Published:** 2013-03-31

**Authors:** Soren Gantt, William J. Muller

**Affiliations:** ^1^Department of Pediatrics, Division of Infectious Diseases, University of Washington and Seattle Children's Hospital, Seattle, WA 98105, USA; ^2^Department of Pediatrics, Division of Infectious Diseases, Northwestern University Feinberg School of Medicine, Chicago, IL 60611, USA

## Abstract

Herpes simplex viruses types 1 and 2 (HSV-1 and HSV-2) infect a large proportion of the world's population. Infection is life-long and can cause periodic mucocutaneous symptoms, but it only rarely causes life-threatening disease among immunocompetent children and adults. However, when HSV infection occurs during the neonatal period, viral replication is poorly controlled and a large proportion of infants die or develop disability even with optimal antiviral therapy. Increasingly, specific differences are being elucidated between the immune system of newborns and those of older children and adults, which predispose to severe infections and reflect the transition from fetal to postnatal life. Studies in healthy individuals of different ages, individuals with primary or acquired immunodeficiencies, and animal models have contributed to our understanding of the mechanisms that control HSV infection and how these may be impaired during the neonatal period. This paper outlines our current understanding of innate and adaptive immunity to HSV infection, immunologic differences in early infancy that may account for the manifestations of neonatal HSV infection, and the potential of interventions to augment neonatal immune protection against HSV disease.

## 1. Introduction

Young infants are highly vulnerable to infections due to changes that occur in the immune system during the transition from fetal to postnatal life [1–3]. Herpes simplex virus (HSV) infection exemplifies this paradigm [4]. When HSV infection occurs within the first several weeks of life, the majority of infants will die without treatment. However, acquisition of HSV after this period is typically mild or even asymptomatic. In this paper, we explore what is known about mechanisms by which immunologic control of HSV infection may be impaired in early infancy. Furthermore, we discuss the implications of these findings for developing interventions to better prevent and treat neonatal HSV infection and suggest directions for future research.

## 2. Herpes Simplex Virology and Pathogenesis

The herpes simplex viruses (HSV-1 and HSV-2) are members of the neurotropic *α*-herpesvirus subfamily of the *Herpesviridae* family of viruses (reviewed in [5, 6]). This family includes a variety of enveloped, icosahedral capsid-containing, linear double-stranded DNA viruses with relatively large genomes, many of which cause diverse diseases in humans. All members of the family share the capacity to remain latent in the infected host and are capable of periodic reactivation and spread to new hosts.

Outside the newborn period, primary infection in immunocompetent individuals may cause gingivostomatitis, pharyngitis, or ulcerative genital lesions [7], but infection is frequently subclinical [8, 9]. In most chronically infected individuals, reactivation of virus is either asymptomatic or at most leads to bothersome mucosal lesions [10].

Spread of HSV within the population generally results from reactivation of virus from latently infected neurons within sensory ganglia and anterograde axonal transport to the innervated mucosa, with subsequent viral replication in the epithelium and shedding [11]. The grouped vesicular and/or ulcerative lesions typical of HSV may or may not occur during these episodes, and subclinical genital shedding of HSV-2 is as common in those without any history of genital lesions as it is in those with such a history [12]. Episodes of asymptomatic genital shedding appear to decrease over time, with reactivation occurring more than twice as often in the first three months after primary first-episode HSV-2 genital infections than in subsequent three-month periods [13]. However, short bursts of asymptomatic viral reactivation occur surprisingly frequently for both oral and genital HSV, with about half of genital mucosal reactivations lasting less than 12 hours and more than 70% of these episodes occurring without symptoms [14].

## 3. Epidemiology and Clinical Manifestations of Neonatal HSV Infection

HSV infections are common, with seroprevalences in American adults of about 60% for HSV-1 and 17% for HSV-2 [15]. HSV generally initiates infection at mucosal surfaces and spreads along sensory neurons to establish latency within ganglia (typically the trigeminal or sacral ganglia) [16]. Neonatal HSV infection (defined as occurring before 28 days after birth) occurs in between 1 in 12,500 and 1 in 1,700 live births in the United States, which in combination with its high morbidity makes it a major public health concern (reviewed in [4]). Less than half of neonatal HSV infections occur in the setting of long-standing maternal infection, with a risk of transmission of <1% even when virus is detectable in the maternal genital tract at the time of delivery. In contrast, when a woman acquires HSV late in pregnancy, the risk of neonatal HSV is 25%–50%; this scenario accounts for about 50%–80% of all cases of neonatal HSV [4]. The difference in transmission risk between women with established and recent HSV infections suggests the importance of transplacental maternal antibody [17–21], as discussed in greater detail later, as well as the higher viral titers present during primary maternal infection. 

HSV-1 is commonly associated with oral mucosal infection and HSV-2 with genital infection. However, genital infection with HSV-1 is increasing in prevalence, with recent studies suggesting it surpasses HSV-2 as a cause of genital infection in several different populations [22–29]. It has been speculated that this observation is related to a recent trend of acquiring oral HSV-1 infection later in life along with an increase in oral sex in young adults; this results in a population susceptible to genital HSV-1 infection at initiation of sexual activity [30]. Importantly, several studies suggest that both symptomatic and asymptomatic genital shedding of HSV-1 is less frequent than that of HSV-2 [13, 31–33], which may have implications for neonatal infection.

### 3.1. *In Utero* Infection

Intrauterine HSV infection is associated with hydrops fetalis and fetal death. Surviving infants of *in utero* HSV infection have symptoms at birth similar to other congenital infections, including microcephaly, hydranencephaly, chorioretinitis, and rash, although the presentation is highly variable [18, 79]. Although it is highly morbid, *in utero* HSV infection accounts for <5% of all neonatal cases, or approximately 1 per 250,000 deliveries [80, 81]. It is unclear why fetal infection occurs so infrequently, for example, compared to cytomegalovirus, but multiple factors may be involved [82]. First, detection of HSV DNA in peripheral blood, though relatively common during primary infection, is rare during established disease, even with clinical reactivations [83]. Second, the maternal-placental interface appears to have fairly effective mechanisms to block the spread of HSV. Interestingly, even in cases with severe or disseminated maternal HSV infection during pregnancy, the fetus is often spared [84]. HSV DNA can be detected by PCR in a surprising proportion of placentas (roughly 10%) [85, 86]. Immunohistochemical detection of HSV antigen is less common in placentas, but small foci of virus may be detected in the maternal decidua adjacent to the placenta [86].

### 3.2. Intrapartum and Postpartum Infection

The majority (roughly 85%) of neonatal HSV infections are acquired during passage through the birth canal [19, 87]. Only approximately 10% of neonatal HSV infections occur in the postpartum period, generally through contact with virus shed by caregivers, and these are typically caused by HSV-1. Infection of the newborn is thought to occur through mucosal (eyes, mouth) or cutaneous inoculation.

Neonatal HSV disease is classified into three clinical syndromes: localized skin, eye, and mouth (SEM); central nervous system (CNS) involvement with or without SEM; disseminated disease, which involves spread to visceral organs. Disseminated disease may or may not involve the CNS and can lead to hepatitis, pneumonitis, disseminated intravascular coagulation, shock, and multiple organ dysfunction syndrome. SEM disease accounts for approximately 45% of neonatal HSV cases, though these infants often progress to CNS or disseminated disease in the absence of treatment. CNS and disseminated disease represent approximately 30% and 25% of cases, respectively. Either HSV-1 or HSV-2 can cause SEM, CNS, or disseminated disease, although CNS infection with HSV-2 has been associated with greater morbidity [4, 88–90]. Newborns with severe HSV infection frequently present without fever or skin lesions. Delayed diagnosis and initiation of therapy occur often and contribute to poor outcomes [88]. Even with optimal treatment (60 mg/kg/day of intravenous acyclovir for 21 days), mortality is still approximately 6% with CNS disease and >30% with disseminated disease [4]. Furthermore, treatment has had little effect on neurologic morbidity among survivors of CNS disease [91]. Encouragingly, recent studies have shown that suppressive oral antiviral therapy can improve long-term neurologic outcomes after CNS disease; however, abnormal neurologic outcomes were still reported for 31% of newborns with a history of HSV encephalitis who received suppressive treatment [92]. Thus, additional strategies are needed to better prevent and treat neonatal HSV infection.

## 4. Immune Control of HSV Infection

Despite intense study, our understanding of immunologic control of primary and recurrent HSV infections in humans remains incomplete. Virtually every aspect of immune defense appears to be involved in control of HSV infection, from antimicrobial peptides (AMPs; reviewed in [93]) through the intrinsic antiviral responses of infected cells (reviewed in [94]), innate immune effector cells and cytokines [95, 96], adaptive cellular responses [97, 98], and humoral responses [99]. In immunocompromised humans, defects in multiple arms of the immune response have been described as leading to severe HSV disease, including deficiencies in AMPs [100, 101], various defects in signaling (including signals mediated by TLR3 [102–107], STAT1 [108], tyrosine kinase 2 [109], and NF-*κ*B [110]), other mutations affecting lymphocyte function [111], and abnormalities in numbers or function of NK cells [49, 50], plasmacytoid dendritic cells (pDCs) [112, 113], and T cells [97].

Studies in animal models of HSV infection have shed light on the interaction of innate and adaptive immunity in the response to primary infection. The pattern recognition receptors (PRRs) that have been reported to recognize HSV include TLR2, TLR3, TLR9, the RIG-I-like receptors (RLRs) RIG-I and melanoma differentiation-associated gene 5 (MDA5 or interferon (IFN) induced with helicase C domain 1 (IFIH1)), NOD-like receptors (NLRs), interferon-inducible protein 16 (IFI16), the helicase Ku70, DNA-dependent activator of IFN-regulatory factors (ZBP1), and the helicases DEAH box 9, DEAH box 36, and DDX60 (reviewed in [43, 44]). HSV is capable of infecting monocyte-derived dendritic cells (DCs), including Langerhans cells in the skin and vaginal mucosal epithelia, inducing partial maturation but ultimately leading to apoptosis [114]. Migratory submucosal or dermal DCs phagocytose apoptotic debris, including HSV antigen, and migrate to draining lymph nodes [115, 116]. These cells then appear to either transfer antigen to resident DCs within the lymph node for priming of effective CD8^+^ T cell responses [117] or in some situations will themselves contribute to priming of CD4^+^ and CD8^+^ responses [118].

T cell responses against HSV in humans are polyfunctional [119] and directed against a wide array of viral epitopes [120]. Infiltration of HSV-specific T cells into infected tissue initially involves CD4^+^ T cells, which in mice are required for subsequent CD8^+^ T cell entry into the mucosa [121]. Local NK cells likely contribute to control of HSV replication early in infection and make some IFN-*γ* in the infected tissue [51], but IFN-*γ* from infiltrating CD4^+^ T cells and production of CXCR3-dependent chemokines (likely by epithelial cells) are required for CD8^+^ T cells to efficiently enter the vaginal mucosa [121]. The chemokine gradient required for proper migration of T cells to the site of infection is coordinated in part by regulatory T cells (Tregs) [75].

HSV-specific CD8^+^ T cells appear to be the central effectors controlling latent HSV infection in neurons. In mice, activated HSV-specific CD8^+^ T cells are retained in latently infected sensory ganglia, blocking viral reactivation through IFN-*γ* production without killing the neurons [122, 123]. A reduction in these cells can be seen in conditions of stress, leading to viral reactivation [124]. Recurrence of HSV lesions in skin and mucosa after reactivation from latency also activates local NK cell responses and memory CD4^+^ T cells, followed by infiltration of virus-specific CD8^+^ T cells, in a manner similar to primary infection [125, 126]. Coordination of cellular responses to HSV reactivation in the skin and mucosa is also largely mediated by DCs, in conjunction with B cells [127, 128]. Memory CD4^+^ T cells in mice are restimulated to produce IFN-*γ* by local MHC-II^+^ DCs and B cells [127]. Memory CD8^+^ T cell responses are also initiated by tissue-resident DCs, without requiring DC migration to draining lymph nodes [128]. In humans, infiltrating virus-specific CD8^+^ T cells persist at the dermal-epidermal junction for weeks after virus has been cleared, localizing to peripheral nerve endings [56]. Evidence suggests that these cells may be frequently exposed to viral antigen even in the absence of lesions [129], consistent with observations of frequent short bursts of asymptomatic HSV shedding at mucosal surfaces [14]. Modeling studies based on human data suggest that the local immune response is the critical determinant of genital HSV-2 shedding episodes and the development of lesions [57].

In the central nervous system, innate immune signaling through the TLR3 pathway is clearly important in controlling HSV replication during both primary infection and recurrence. Humans with specific defects affecting TLR3 signaling have increased susceptibility to encephalitis, with mutations described in UNC93B [103], TRAF3 [104], TRIF [105], and TLR3 [106, 107]. In mice, TLR2 signaling appears to be important in controlling HSV replication in the brain [130]. Interestingly, however, TLR2 has also been described as contributing to lethality of mice with HSV infection in the central nervous system by dramatically increasing the inflammatory response [131], in a manner regulated by the surface glycoprotein CD200R1 [132]. This concept that CNS inflammation can promote pathogenesis in HSV encephalitis is supported by other murine studies [65–67, 133].

Given that the human immune system directs multiple varied mechanisms at detection and control of HSV infection, it is not surprising that the virus allocates a significant proportion of its genome to overcoming the anti-HSV immune response. HSV modulation of immune responses essentially begins from the time the virus encounters a susceptible cell. Engagement of the HSV entry receptor known as herpes virus entry mediator (HVEM) modifies expression of a number of cellular genes, which may immediately alter the cell environment to promote viral replication [134] or alter mucosal chemokine and cytokine production [135]. These consequences of the HSV-HVEM interaction are thought to be due to transient NF-*κ*B activation, which at later times after infection may promote viral gene expression in addition to modifying expression of cellular targets of NF-*κ*B [136]. Subsequently, the viral “virion host shutoff” protein (vhs), which is delivered to the cell within the tegument of the viral particle, promotes degradation of cellular mRNA, inhibiting synthesis of a variety of inflammatory proteins including cytokines and type I IFNs [137]. Additional proteins are expressed relatively early in infection to target different intrinsic antiviral cellular responses, inhibiting various proinflammatory and proapoptotic signaling proteins such as PKR and IRF3, and inhibiting type I IFN signaling pathways by mechanisms such as repression of STAT1 activation [94]. Other innate and adaptive immune responses are also targeted by HSV proteins, including binding of complement by the glycoprotein C (gC) [138, 139], binding of the Fc domain of IgG by the gE/gI complex [140], and interference with TAP-mediated peptide loading onto class I MHC by ICP47 [141–143]. 

In addition to suppression of cellular production of antiviral proteins, recent work suggests that other cellular processes intended to inhibit viral replication are targeted by HSV. There has been increasing appreciation of the importance of autophagy in resistance to HSV infection [144], particularly in neurons. HSV needs to evade autophagy to cause encephalitis [38], and the virus encodes at least two proteins which target this process. HSV ICP34.5 protein binds and inhibits the cellular autophagy protein Beclin-1 to promote neurovirulence [39], and US11 directly binds and inhibits the double-stranded RNA-dependent kinase PKR, which functions in induction of the autophagic response [40]. Importantly, neurons, in contrast to mucosal cells, require autophagic activity to limit HSV replication but do not respond effectively to stimulation by type I IFN [145].

## 5. Aspects of HSV Immunity Specific to Neonates

Human fetuses and newborn infants are more susceptible to severe infection with a wide variety of different pathogens compared to older children or adults. Fetal and neonatal immune responses have long been recognized to have qualitative differences that change during infancy, and we are beginning to understand the mechanisms that underlie this process (reviewed in [1–3]). The immunology of the young infant is dynamic and complex; for example, some innate responses become more “adult-like” within weeks, while others take a year or more [3, 146–149]. Beyond several weeks of life, acquiring HSV portends virtually none of the severe risks of neonatal infection [150]. In comparison, the risk of progression from tuberculosis infection to active disease remains elevated until approximately 4 years old [149, 151]. 

Fetal immunology likely represents an evolutionary strategy that contributes to successful parturition through maternofetal tolerance. The fetus and its mother are haploidentical, and thus have the potential for alloreactive responses akin to rejection. Inflammation is generally harmful to the developing fetus, resulting in a number of adverse outcomes including intrauterine growth retardation, premature birth, and spontaneous abortion (reviewed in [152]). The mechanisms that underlie maternofetal tolerance remain incompletely understood but include differential expression of class I HLA molecules [153], altered NK cell activity [154], increased numbers and suppressive activity of regulatory T cells (Tregs) [155–159], myeloid-derived suppressor cells (MDSCs) [160], high levels of adenosine [2] and progesterone [161, 162], and differences in TLR responses [45, 46]. Some or all of these mechanisms may impact immune responses during postnatal life and contribute to the vulnerability of neonates and young infants to infection, as well as to vaccine responses that are generally inferior to older children [1, 2, 46, 61]. Here we focus on those aspects of immune ontogeny with apparent importance for neonatal HSV infection (Table 1). 

### 5.1. Skin Barrier Function

Differences in epithelial mechanical integrity and production of AMPs may contribute to increased HSV severity in neonates. The epidermis of the fetus and newborn is thinner than that of an adult, predisposing to disruption by trauma [34]. Skin disruption likely increases the risk of neonatal HSV infection, given the association between invasive monitoring (scalp electrodes) and neonatal HSV infection [163]. Other aspects of skin also develop during the neonatal period, including acidification and production of sebum lipids, and might affect HSV infection or replication. Although in theory maternal- and fetal-derived AMPs may help prevent HSV infection by blocking entry and replication of virus on mucocutaneous epithelial surfaces, there is no evidence that differences in newborn AMP expression or activity contribute to HSV disease. AMPs such as cathelicidin and lysozyme are abundant in amniotic fluid, vernix caseosa, and newborn epithelia [164–167], and levels of some AMPs appear to be elevated in neonates compared to later in life [35–37]. Nevertheless, identifying and augmenting barrier host defenses may have potential to protect against HSV acquisition or reduce disease severity.

### 5.2. Autophagy

 It is unclear if differences in autophagy in neonatal central neurons relative to older children and adults affect the severity of HSV infection. However, this would not be surprising given rapid growth and development of the brain during this period and the role of autophagy in neurodevelopment [168, 169]. In addition, autophagy can be induced by signaling through several TLRs associated with control of HSV in the nervous system, including TLR2, TLR3, and TLR9 [41, 42]. As discussed later, there are differences between neonates and adults in the effects of TLR signaling on conventional innate immune responses; similar developmental differences may exist with respect to TLR induction of autophagy.

### 5.3. Pattern Recognition Receptor Mediated Responses

TLR responses change profoundly with age (reviewed in [46]). The expression and function of other PRRs (including RLRs, NLRs, IFI16, etc.) during the neonatal period have not been as well described but are also likely to change during early life [170] and may be important in the pathogenesis of neonatal HSV. The involvement of TLR signaling in protection (TLR3) or pathologic inflammation (TLR2) during HSV CNS disease suggests the possibility that developmental differences in TLR responses may be involved in the susceptibility of neonates to severe HSV infection. Compared to adults, conventional DCs from cord blood produce significantly less IFN-*α* and IL-12 upon stimulation with the TLR3 agonist poly(I:C) and show lower expression of CD40 and CD80 [171]. Indeed, IFN-*α* and IL-12p70 (and consequently IFN-*γ*) responses to most TLR agonists, including PAM_3_CSK_4_ (TLR2/6) and CpGA (TLR9), appear to be relatively weak in conventional and plasmacytoid DCs as well as monocytes from cord blood, while IL-1*β*, IL-6, IL-23, and especially IL-10 responses are as high or often much higher than in adult PBMC [45]. Experiments using neonatal mice found improved control of HSV-1 infection by expanding the number of DCs with Flt-3 ligand (Flt3-L) treatment, which resulted in increased production of IFN-*α*/*β* and IL-12 [71]. Consistent with these findings, IFN-*α* production in cord blood or neonatal mononuclear cells appears to be reduced in response to *in vitro *HSV-1 stimulation [18, 172]. This pattern of a neonatal bias, toward Th2- and Th17-type and away from Th1-type responses, is consistent with impaired control of HSV infection (as well as other intracellular pathogens), and perhaps with increased pathologic inflammation [46]. Interestingly, the ontogeny of individual TLR responses varies. Stimulation of monocytes using TLR3 agonists leads to lower levels of IFN-*α* when cells isolated from children age 1 or lower are compared with those from adults; however, for TLR9 agonists, comparable responses can be demonstrated within the first few weeks of life [47, 146], that is, the same time period during which infants are susceptible to severe HSV infection.

The mechanistic basis for differences in TLR responses in neonates is not well understood [46], but preliminary evidence suggests that both cell-cell interactions and soluble blood factors may be involved [45, 173]. Decreased MyD88 expression in neonatal monocytes [174, 175] might explain some of these effects given that this adaptor protein is utilized by all TLRs, with the exception of TLR3. Of note, adult MyD88-deficient mice do not control HSV after corneal inoculation and progress to fatal encephalitis [176]. However, MyD88 expression was reported to be equal in purified neonatal and adult pDCs, while reduced type 1 IFN production in neonatal pDCs appeared to be due to impaired nuclear translocation of IRF7 [177]. Decreased IFN-*β* production may be due in part to altered DNA binding of CREB and IRF3 [178]. Developmental differences in nucleosome remodeling and availability of cytokine promoter sites, for example, IL-12p35 [179, 180], in neonatal monocytes and antigen-presenting cells may be responsible for some of observed cord blood TLR responses, similar to what has been reported for IFN-*γ* in T cells [181]. Increased levels of adenosine, which increases intracellular cAMP [182], as well as other soluble factors [173] in neonatal blood, also appear to be important for suppressing Th1-polarizing responses. 

### 5.4. Natural Killer Cells

Impaired killing of HSV-infected cells by cord blood mononuclear cells has long been recognized [52–54]. This could result from impaired activation by cytokines such as type 1 IFNs and IL-12, the production of which is reduced in neonates as mentioned earlier, or may reflect intrinsic differences of neonatal NK cells. NK cells in cord blood may differ with respect to the expression of cell surface markers compared to those from the peripheral blood of adults (reviewed in [55]). For example, some studies have found higher levels of the inhibitory receptor complex of CD94/NKG2A and CD158b/j on cord blood NK cells [183, 184]. However, NK cell IFN-*γ* production in response to mitogen appears to be similar between cord blood and adult peripheral blood [183, 185]. Several studies showed that resting cord blood NK cells are less cytotoxic; however, these cells may actually express higher levels of effector molecules such as perforin and granzyme B [183, 186] and can be induced to be highly cytotoxic using stimulation with various combinations of IL-2, IL-12, IL-15, and IL-18 [183, 187–190]. Thus, any impairment in neonatal NK function may represent extrinsic factors, for example, deficient IL-12 production by DCs. The extent to which any NK cell differences in cord blood are relevant to neonatal HSV infection is unknown, but it merits additional study given that NK cells likely play an important role control of HSV infections in general [49, 50, 191, 192].

### 5.5. Adaptive T Cell Responses

Early studies of neonatal T cell responses to HSV suggested that newborns generate fewer virus-specific cells, which have impaired proliferation to stimulation with virus [58–60]. Furthermore, IFN-*γ* production in response to HSV antigen was significantly lower among neonates and parturient women compared to nonparturient adults, all with recent HSV acquisition [60]. In that study, HSV-specific neonatal IFN-*γ* responses lagged behind those of nonparturient adults until 3–6 weeks after the onset of symptoms. Numerous differences have been described between general responses of neonatal T cells relative to those of adults [2, 61, 62]. Fetal T cells appear to be derived from a distinct lineage compared to adult T cells and are biased toward tolerance [193]. Compared to those from adults, neonatal CD4^+^ T lymphocytes are more apt to produce Th2- than Th1-type cytokines under the same conditions [194, 195]. Newborn CD4^+^ T cells may produce lower levels of IFN-*γ* than adult naïve T cells due to hypermethylation at CpG and non-CpG sites within the IFN-*γ* promoter [181, 196]. Interestingly, neonatal CD8^+^ T cells produce similar levels of IFN-*γ* and have a pattern of IFN-*γ* promoter methylation comparable to that of naïve adult cells [196]. In addition, neonatal cellular responses may be inhibited by the presence of suppressive soluble factors or suppressor cell populations. 

Substantial evidence suggests that regulatory T cells (Tregs) play a critical role in maternofetal tolerance [155–159]. Studies in mice suggest that Tregs may also differentially suppress neonatal CD8^+^ T cell responses to HSV compared to adults [197]. Other suppressor cell populations may be involved in neonatal immunity. MDSCs are heterogeneous populations of immature granulocytes or monocytes that suppress T cell responses and are important in tumor immunology (reviewed in [198–200]). MDSCs appear to prevent inflammation *in utero *based on studies in mice [160]. Although they are present in peripheral blood in very small numbers in healthy adults, MDSCs are found in high frequencies in pregnant women and cord blood and wane during infancy (Helen Horton, personal communication). MDSCs have been reported to preferentially induce Th-2 responses and impair NK and DC responses (reviewed in [201–203]), all of which are characteristic of neonatal immune responses. However, the extent to which Tregs, MDSC, or other suppressor cells contribute to susceptibility to HSV or other infections during postnatal life requires additional research.

### 5.6. Antibody

As discussed earlier, virus-specific antibody might prevent HSV acquisition via neutralization or contribute to control of infection through neutralization or ADCC. A large number of observational studies suggest that, though not completely protective, maternal anti-HSV antibody can reduce the risk of neonatal HSV acquisition [17–21]. This is also supported by studies in mouse models showing protection against neonatal HSV infection by virus-specific maternal antibodies [204–206]. High titers of antibody to HSV among infected newborns at presentation have been suggested to result in less severe disease in some studies [18, 207] but not others [208, 209]. Infants typically develop virus-specific IgM within weeks of HSV infection; however, there is no evidence that these responses contribute to control of infection, recurrences, or outcome. This is consistent with the apparent lack of association between humoral immunodeficiencies and severe HSV infections.

## 6. Possible Interventions Targeting Host Defenses to Prevent Neonatal HSV

### 6.1. Vaccination

Vaccination is considered the intervention with the greatest potential for preventing neonatal HSV [4]. Since acquisition of primary infection during pregnancy confers the highest risk for neonatal HSV disease [163, 210], a prophylactic vaccine would ideally confer high levels of protection against genital HSV in pregnant women. Despite extensive efforts, there are currently no licensed vaccines to either prevent HSV acquisition or minimize transmission in humans [63, 211]. Clinical trials of candidate prophylactic vaccines against HSV-2 have demonstrated limited clinical activity [212–214]; however, these studies have uncovered important information about host immunity to HSV. Randomized trials of a subunit vaccine comprised of the HSV-2 surface glycoproteins gB and gD revealed that serum neutralizing antibody levels did not correlate with protection from HSV infection in humans [213], suggesting that additional responses, perhaps mucosal or cellular responses, are also needed to confer sterilizing immunity [215]. Even in the absence of complete protection against HSV infection, a prophylactic vaccine that modified the course of infection, by limiting maternal viral reactivation and genital shedding, could still reduce neonatal disease. Such a vaccine would likely require induction of cellular immune responses to HSV. Indeed, a prior study of therapeutic vaccination of individuals with latent genital HSV-2 infection suggested that recurrences might be diminished in some individuals in a manner that did not correlate with antibody production, supporting the concept that cellular immunity is critical to protecting the mucosa from HSV replication [216]. From this standpoint, recent studies elucidating the mechanism of infiltration of HSV-specific CD8^+^ T-cells into genital mucosa [121] may provide insight into novel vaccination strategies, such as the “prime-pull” strategy suggested by Shin and Iwasaki [217].

Alternative approaches to improve vaccine responses have aimed to enhance immunogenicity. Among those showing preclinical promise in animal models are novel delivery systems such as liposomes [218], modified recombinant bacteria expressing HSV antigens [219], and incorporating the use of novel adjuvants [220] or DNA vaccines [221, 222]. One possible limitation of these approaches is their reliance on a single or a limited set of viral antigens (typically gD, often with 1-2 additional targets) to provide protection against a virus with a genome that encodes more than 80 proteins. Broader antiviral responses could be generated with attenuated or replication-defective viral vectors [215, 223, 224], which in some instances have demonstrated protection in animal models [225–228]. However, it is not clear whether some of these alterations of the virus may remove important targets of human immunity. Other modifications to the viral genome, such as inserting a dominant-negative mutant gene [229] or inserting costimulatory genes [230, 231], may carry the risk of gene transfer to a wild-type virus through heterologous recombination [232]. Novel delivery systems or adjuvants need to be produced in a cost-effective manner, require careful evaluation for safety in humans, and may promote unacceptable inflammatory responses [233].

### 6.2. Other Immunologic Strategies to Prevent HSV Infection

 Biological products based on immune proteins, such as cationic AMPs, have been proposed as potential candidates to protect against HSV infection [93, 234, 235]. These molecules might be formulated as microbicides, for example, to prevent maternal acquisition of HSV, or transmission to the infant during birth [64].

## 7. Possible Interventions to Modulate the Immune Response to Neonatal HSV Infection

Alternative approaches to modify host immunity for treatment of neonatal HSV infection have been suggested (Table 2). These strategies have hypothetical benefits that merit study but should not be considered for clinical use until safety and efficacy have been established. 

### 7.1. Suppression of Local Inflammatory Response in the CNS

As discussed earlier, data from animal models suggest that deleterious inflammatory responses may play an important role in the pathogenesis of HSV encephalitis [65–67]. Numerous cases of HSV encephalitis treatment using adjunctive corticosteroids with good outcomes, mostly in adults, have been reported [68–70]; however, given the risk of increased viral replication and cytotoxic effects, this approach is controversial [236, 237]. In order to develop targeted immunomodulatory therapies for neonatal HSV infection, a better understanding is needed of the relative contributions and temporal dynamics of the specific inflammatory pathways that mediate control of viral replication and immune-mediated CNS damage [238].

### 7.2. Targeting Autophagy

Development of novel antivirals has been proposed to target HSV proteins that inhibit autophagy [76], and early studies suggest that agents that induce autophagy can inhibit HSV replication [77, 78]. 

### 7.3. Promotion of Th1-Type Responses

 Strategies can also be envisioned that promote Th1-type responses during the neonatal period through novel adjuvants like TLR agonists [47, 48], growth factors such as Flt3-L [71], or other agents that target antigen-presenting cells. If suppressor cell populations are confirmed to impair neonatal immune responses, interventions to oppose the effects of these cells might result in Th1-type responses to HSV more similar to those produced by adults. In models of other diseases, such as HIV infection and melanoma, therapies have been proposed to reverse Treg activity and enhance protective T cell responses, for example, with recombinant IL-7 or blockade of negative costimulatory receptors CTLA-4 and PD-1 [72, 73]. Similarly, studies in solid tumor patients have shown that the suppressive activity of MDSC can be reversed by 25-hydroxyvitamin D_3_, all-trans-retinoic acid, and other therapies [74]. Any of these immunomodulatory strategies might be expected to improve neonatal responses not just to HSV, but also other neonatal pathogens and vaccines, as well as to potentially prevent atopic diseases [48]. It should be stressed again that any benefit of these interventions for neonatal HSV infection is currently entirely theoretical, and their use for any indication requires extensive study to assure safety in newborns.

## 8. Conclusions and Directions for Future Research

An effective prophylactic HSV vaccine represents an ideal way to prevent neonatal HSV infection. In the absence of such a vaccine, early recognition and aggressive antiviral treatment of neonatal HSV infection remain the mainstays of care. The development of new interventions for neonatal HSV discussed earlier requires a better understanding of the mechanistic basis of immune control of HSV infection in general and how neonatal responses to HSV are ineffective by comparison. Specifically, more studies are required to understand the basis of differential TLR and other pattern recognition receptor responses in early life and their effects on neonatal HSV infection. Differences in T cell responses to HSV between neonates and older children or adults also merit more study, as do the relative contributions of impaired priming, inherent differences in T cell signaling, and/or active suppression on poor cellular control of HSV infection during the newborn period. The role of Tregs, MDSC, and other suppressor cell populations in immune control of HSV infection is also of great interest. More complete knowledge of immune ontogeny could lead to interventions that might be routinely given to all newborns to improve immune responses not just to HSV, but to a wide range of other infectious pathogens and vaccines as well. 

Just as important as understanding the immunology of how neonates differ from older children and adults, however, is to determine what benefits if any there are during early postnatal life that come from the apparent persistence of *in utero* tolerance. If immune response patterns in early infancy simply represent a transition between fetal and adult-type immune responses that requires time but serves no function, it may be safe and advantageous to expedite this process. It is possible, however, that the ontogeny of immune system during early postnatal life is evolutionarily adaptive. It has been hypothesized that without relative tolerance immediately postpartum, rapid colonization of newborns with myriad microorganisms and non-self-antigens might lead to overwhelming inflammation [2]. Other possibilities, which are not mutually exclusive, include the possibility that relative neonatal tolerance protects against autoimmunity and allergies [170, 239]. Neonatal HSV infection represents both an important clinical problem and a fascinating example of age-dependent immunity. Through a greater understanding of the dynamic interplay between the virus and host, there are opportunities to rationally develop safe and effective therapies to prevent or treat neonatal HSV infection.

## Figures and Tables

**Table 1:** Immune defenses against HSV with relevance for neonates.

Immune defense	Role in controlling HSV infection	Immunologic differences in newborns	Comments
Integument	The skin and mucosa provide mechanical and innate antiviral impediments to HSV infection and spread.	Neonates have thin, easily disrupted skin, with differences in pH and sebum production [34].	Differences in neonatal epithelial anatomy or function have not been formally shown to contribute to susceptibility to HSV infection. Levels of some AMPs appear to be increased during the neonatal period [35–37].

Autophagy	HSV-mediated suppression of autophagy is central to the pathogenesis of CNS infection [38–40].	Autophagy is mediated by signaling through TLRs, which have age-dependent responses [41, 42].	Age-dependent differences in autophagy are plausible but poorly understood.

PRR responses	PRR signaling in HSV-infected cells induces type 1 IFN production that limits initial spread of infection through and attracts and primes protective Th1-type responses [43, 44].	Neonates have qualitatively different monocyte and DC TLR responses that result in reduced type 1 IFN and IL-12 production, resulting in weaker Th1-type responses [45–48].	Age-dependent TLR3 responses to HSV are likely important based on the association between CNS HSV infections and defects in TLR signaling. Age-dependent effects of other TLR or PRR responses are unclear but may also be important for the severity of HSV infection in neonates.

NK cells	NK cells are important for control of initial HSV infection prior to development of specific T cell responses [49–51].	Neonates appear to have impaired NK cell killing of HSV-infected cells [52–54].	Whether neonatal NK cells have any intrinsic defects or kill less well as a result of impaired activation, for example, decreased IL-12 production by DCs, is unclear [55].

T cell responses	CD8^+^ T cell responses appear central to control of HSV replication and prevention of recurrence [56, 57].	Neonatal T cells respond relatively poorly to HSV [58–60].	Impaired Th1-type responses against HSV in neonates may be due to differences in innate responses by antigen-presenting cells, intrinsic epigenetic factors (e.g., hypermethylation of the IFN-*γ* promoter in CD4^+^ cells), or perhaps active suppression by suppressor cells [2, 61, 62].

Antibody	HSV neutralizing antibody or ADCC may protect against acquisition of infection [17–21].	Infants born to women with established HSV infections receive virus-specific transplacental maternal antibody [4].	Although infants of women with established HSV infection are much less likely to become infected compared to those who acquire primary infection during pregnancy, no definitive proof exists that antibody alone is protective in humans. After infection, antibody responses do not appear to contribute significantly to control of HSV replication.

**Table 2:** Potential interventions targeting host defenses against neonatal HSV infection.

Potential intervention	Comments
Maternal vaccination	No effective HSV vaccine is yet available. Neonatal HSV infection could be prevented by a vaccine that either conferred sterilizing immunity to women prior to pregnancy and/or by modifying infection in women to reduce viral replication and shedding in the genital mucosa [63].

Antimicrobial peptides	AMPs formulated as a vaginal microbicide might prevent HSV infection during pregnancy and/or reduce intrapartum transmission [64].

Immunosuppressive therapy for CNS infection	Some component of the immune response to HSV encephalitis may result in pathologic inflammation and contribute to poor outcomes [65–67]. Despite case reports of good outcomes using adjunctive corticosteroids in adults or neonates with HSV CNS infection [68–70], no controlled studies have been performed and this or other immunosuppressive treatments cannot currently be recommended given the risks of increased viral replication and cytotoxic effects.

Immunomodulation of neonatal Th2/Th17 bias	Th1-type responses might be promoted during the neonatal period with novel adjuvants such as imazoquinolines [47, 48], growth factors such as Flt3-L [71], or other agents that target antigen-presenting cells. These strategies might conceivably be used therapeutically during infection or to prime all neonates to respond to infection and vaccinations [48].

Inhibition of suppressor cell function	Tregs, MDSCs, or other suppressor cell populations might contribute to impaired T cell responses during early infancy. Modulation of these cells' activity might improve immunity to HSV infection, such as what has been proposed for HIV and cancer [72–74]. Inhibition of suppressor cell function during HSV infection might also result in uncontrolled inflammation and worse outcomes [75].

Induction of autophagy	Novel antivirals have been proposed to target HSV virulence factors that inhibit autophagy [76], and early studies suggest that agents that induce autophagy can inhibit HSV replication. Nelfinavir and pentagalloylglucose both induce autophagy and inhibit HSV replication *in vitro* [77, 78].
